# Associations among Bruxism, Gastroesophageal Reflux Disease, and Tooth Wear

**DOI:** 10.3390/jcm7110417

**Published:** 2018-11-06

**Authors:** Yuanyuan Li, Fan Yu, Lina Niu, Wei Hu, Yong Long, Franklin R. Tay, Jihua Chen

**Affiliations:** 1 State Key Laboratory of Military Stomatology & National Clinical Research Center for Oral Diseases & Shaanxi Key Laboratory of Oral Diseases, Department of Prosthodontics, School of Stomatology, The Fourth Military Medical University, Xi’an 710032, Shaanxi, China; YuanyuanLi_1@163.com (Y.L.); fan81811@163.com (F.Y.); niulina831013@126.com (L.N.); weihuta21@163.com (W.H.); longyong@fmmu.edu.cn (Y.L.); 2 Department of Immunology, The Fourth Military Medical University, Xi’an 710032, Shaanxi, China; 3 Department of Epidemiology, School of Public Health, The Fourth Military Medical University, Xi’an 710032, Shaanxi, China; 4 Department of Endodontics, The Dental College of Georgia, Augusta University, 1430, John Wesley Gilbert Drive, Augusta, GA 30912-1129, USA

**Keywords:** tooth attrition, tooth erosion, bruxism, gastroesophageal reflux

## Abstract

The relationship between bruxism and tooth wear is contentious in the literature. The pathophysiological processes of tooth wear may be complicated by the relationship between bruxism and gastroesophageal reflux disease (GERD). The objective of this study was to evaluate the associations among bruxism, GERD, and tooth wear. Two complementary studies were performed: a case-control study to verify the linkage between GERD and bruxism and a cross-sectional study on the same cohort to establish the connection between GERD and tooth wear in bruxism patients. A cohort of 363 consecutive bruxism patients and 363 matched control participants were recruited. Gastroesophageal reflux disease was diagnosed in accordance with the Montreal criteria. Tooth wear was scored based on the index recommended by Smith and Knight. Logistic regression analyses were performed. After adjustment, GERD was identified as a risk factor of bruxism. Bruxism with reflux symptoms for extensive time-periods was associated with severe tooth wear for the whole dentition (odds ratio, 4.70, 95% confidence interval, 2.04–10.83). Increased odds ratios for severe tooth wear were also found in all tooth locations and palatal/lingual and occlusal/incisal surfaces of bruxism patients with GERD for extensive time-periods. In conclusion, strong associations were identified among bruxism, GERD, and tooth wear.

## 1. Introduction

Tooth wear involves at least three courses of action: attrition (wear produced by tooth-tooth contact), erosion (chemical wear caused by acids), and abrasion (wear through tooth-material interaction) [1]. Abfraction is another possible theory that involves occlusal stress in the creation of non-carious cervical lesions [2].

Dentists generally ascribe occlusal wear to attrition [1]. Attrition also leads to wear of palatal/lingual and buccal/labial surfaces, especially with malocclusions [1]. Pathological attrition of occlusal surfaces is commonly associated with bruxism [1]. However, not all studies support the relationship between bruxism and tooth wear [3]. Tooth erosion is a distinctive manifestation of gastric juice entering the mouth in conditions such as gastroesophageal reflux disease (GERD) [1]. Gastric juice has greater erosive effects on both enamel and dentin, compared to extrinsic acids from diet [4,5]. Tooth erosion in GERD patients has also been associated with impaired salivary function [6]. Abrasion is caused by the friction of extrinsic stuff (most commonly food) that is pressed on the tooth surface [7]. These individual wear processes rarely act alone, and usually act together [1,2]. Erosive impact on the tooth tissues renders the surfaces more vulnerable to mechanical wear (abrasion and attrition) and accelerates the processes of tooth wear [1]. When dentin is exposed by expedited wear, erosive agents have even more severe effects on wear processes because dentin is more soluble than enamel [8].

Recent evidence showed that bruxism is associated with GERD [9,10,11]. Mechanisms of tooth wear may be more complicated in bruxism patients considering the relationship between GERD and bruxism. Although a multitude of investigations have probed the association between tooth wear and bruxism or the association between tooth wear and GERD, no study on tooth wear has taken into account the relationship between GERD and bruxism.

Accordingly, the aim of this study was to evaluate if associations exist among GERD, bruxism, and tooth wear. The null hypotheses assessed included: (1) there is no connection between GERD and bruxism; and (2) there is no association between tooth wear and GERD in bruxism patients.

## 2. Experimental Section

This study conforms to the requirements of the Strengthening the Reporting of Observational Studies in Epidemiology [12].

### 2.1. Study Design and Participants

Two complementary studies were performed: a case-control study to verify the linkage between bruxism and GERD and a cross-sectional study on the same cohort to determine if an association exists between GERD and tooth wear in bruxism patients. The study was a supplemental study of our three-center study [13]. The cohort in the present work was recruited from the Stomatological Hospital of the Fourth Military Medical University in China (Xi’an, Shaanxi). The participants recruited in the present work not only met all the criteria of the three-center study [13], but also had eight teeth or more available for tooth wear analysis; the teeth for tooth wear analysis in each participant were opposed by restored or unrestored dentition. Data were collected from August 2017 to March 2018. The ethics committee of the Stomatological Hospital of the Fourth Military Medical University approved the study (ID: IRB-REV-2017028). Informed consent from each participant was obtained prior to the study.

Bruxism patients aged 18–72 years were recruited as cases. One control subject was matched to each case by sex and age (±1 year). Diagnostic criteria for bruxism, subject sources, exclusion criteria for all participants, calculation of study size, and calibration of the dentist have been previously published [13]. In short, diagnostic criteria for bruxism includes patients’ complaint of teeth grinding/clenching when awake or asleep and corresponding manifestations such as tooth wear, locking of temporomandibular joint, and irritation of jaw muscle as defined by the American Academy of Sleep Medicine [13]. The dentist also acted as interviewer in the following interview.

### 2.2. Data Measurement

Data on basic characteristics were gathered. Details have been previously published [13]. Data on occupation, medical conditions, and medication were also collected during the interview. Attention was devoted to identification of the potential factors related to tooth wear. Occupations that may be associated with tooth wear included wine tasters, competitive swimmers, and workers in battery, fertilizer, galvanizing, plating or pharmaceutical factories [14]. Medical conditions that may be associated with tooth wear included hyposalivation-related diseases such as Sjögrens syndrome, rheumatoid arthritis, and eating disorders [14]. Related medications included tranquilizers, antihypertensives, and diuretics, which may cause dry mouth [14].

The intake frequency of acidic foods (fruits: apples, grapes, grapefruit, lemons, oranges; drinks: orangeade, lemonade, lemon juice, orange juice, fruit juice, cola; other acids: vinegar, pickles, salad dressing, curried food) [15] was confirmed from predefined categories (≥6 times/day, 4–5 times/day, 2–3 times/day, 1 time/day, 3–6 times/week, 1–2 times/week, <1 time/week, never or hardly ever). Any recent change in dietary habits was also asked and recorded. Acidic diet was defined as a daily or more consumption of acidic foods. Daily oral hygiene routines were also recorded, including frequency, time, duration, and pattern of brushing or oral hygiene (rinsing or brushing) immediately after exposure to intrinsic or extrinsic acids, type of toothbrush, and chewing and sucking behaviors [16].

We used validated questionnaires to identify subjects with impaired sleep quality, anxiety, depression, and high possibility of obstructive sleep apnea. Diagnosis of GERD was made by one trained and calibrated physician on the basis of the Montreal definition [17]. Details of the questionnaires, GERD diagnosis and calibration of the physician have been previously published [13].

The condition of tooth wear of each participant was scored under ideal lighting using a dental mirror by another trained and calibrated dentist. All teeth were cleaned with distilled water and dried with cotton rolls before examination. Wear on the occlusal/incisal, palatal/lingual, buccal/labial, and cervical surfaces of all existing teeth, excluding the third molars, were scored between 0 and 4 based on the index recommended by Smith and Knight: 0 (no deprivation of superficial properties), 1 (deprivation of enamel superficial properties; minimal deprivation of contour in the cervical areas), 2 (deprivation of enamel, exposing no more than 1/3 of dentin in the buccal, lingual, and occlusal surfaces; deprivation of enamel in the incisal surface just exposing dentin; no more than 1 mm of cervical loss), 3 (deprivation of enamel, exposing more than 1/3 of dentin in the buccal, lingual, and occlusal surfaces; deprivation of enamel and extensive dentin in the incisal surface; 1–2 mm of cervical loss), 4 (exposure of pulp or secondary dentin; more than 2 mm of cervical loss) [18]. The dentist was trained and calibrated at the Stomatological Hospital of the Fourth Military Medical University prior to the acquisition of data. An expert on bruxism played the part of the reference. The reliability studies were performed on 50 volunteers recruited from the hospital (28 with bruxism, 22 without bruxism) on two occasions 14 days apart before data collection. Intra-examiner reproducibility was also evaluated by re-examination of 50 participants randomly selected from the cohort of the present study on two occasions 14 days apart during data collection. The correlation coefficients were higher than 0.95 in all the tests, suggesting good inter-examiner and intra-examiner reliability.

Decayed, missing or restored teeth (restored with any dental prosthesis) were recorded and excluded from analysis. Tooth wear was defined as having at least one surface with a score ≥2, which indicated wear into dentin and/or cervical defect that involved more than the loss of contour. Severe tooth wear was defined as having at least one surface with a score ≥3, which indicated dentin exposure >1/3 of the surface and/or cervical defect ≥1 mm. Calculation was performed for the whole dentition as well as specific tooth surfaces (occlusal/incisal, palatal/lingual, buccal/labial, cervical) and locations (upper, lower, anterior, posterior) of each participant for subsequent analysis.

### 2.3. Efforts to Minimize Bias

All the investigators were trained and calibrated prior to the study to minimize bias associated with differential diagnosis and assessment. The subjects were not informed of the objectives of the research or any possible association among bruxism, GERD, and tooth wear during the study to minimize recall bias. The dentists and the physician were blinded to any participant information that may interfere with their performance. The other efforts to minimize bias have been previously published [13].

### 2.4. Statistical Analysis

We used expectation maximization algorithm to replace missing data. We analyzed categorical variables by chi-squared methods and analyzed continuous variables by Mann-Whitney *U* tests. We conducted logistic regression analysis conditional on age and sex to calculate the odds ratio of bruxism associated with GERD. Potential confounders included residence, education level, marital status, smoking status, alcohol, tea and coffee consumption, depression, anxiety, pain-related temporomandibular disorder, obstructive sleep apnea and sleep quality [13]. The selection of the factors in the multivariable analysis was based on *p*-value <0.1 in the univariate analyses. In the sensitivity analysis, the regression model was further adjusted for tooth wear in addition to other confounding factors because tooth wear was part of the diagnostic criteria of bruxism and may be related to GERD.

We employed unconditional logistic regression analyses to calculate odds ratios of severe tooth wear. Potential confounding factors were identified from the literature [1,14,19] and examined in the univariate analyses: age (<31 years versus ≥31 years, based on the mean age of the cohort), sex (female versus male), smoking (current versus never/former), alcohol (none/moderate versus heavy consumption), tea (weekly or more versus less than weekly consumption), coffee (weekly or more versus less than weekly consumption), acidic diet (no versus yes), frequency of tooth brushing/day (<2 versus ≥2), horizontal tooth brushing (no versus yes), rinsing immediately after exposure to extrinsic/intrinsic acids (no versus yes), brushing immediately after exposure to extrinsic/intrinsic acids (no versus yes), stiffness of bristles (soft/medium versus hard), dry mouth (no versus yes). The selection of the factors in the multivariable analysis was based on *p*-value < 0.1 in the univariate analyses.

Patients were reclassified as non-bruxism, bruxism without GERD, bruxism with GERD ≤5 years, bruxism with GERD >5 years according to the duration of GERD manifestations. We performed unconditional logistic regression analyses to evaluate the associations between severe tooth wear and different GERD duration in bruxism patients. Analyses were performed for severe tooth wear in the whole dentition as well as in different tooth surfaces (occlusal/incisal, palatal/lingual, buccal/labial, cervical) and locations (upper, lower, anterior, posterior). Hosmer-Lemeshow goodness-of-fit tests were performed to evaluate the goodness of fit of each logistic regression model [20]. We performed all the analyses using SPSS 22.0 (IBM Co., Armonk, NY, USA) (two-tailed α = 0.05).

## 3. Results

### 3.1. Case-Control Study

**Figure 1:**
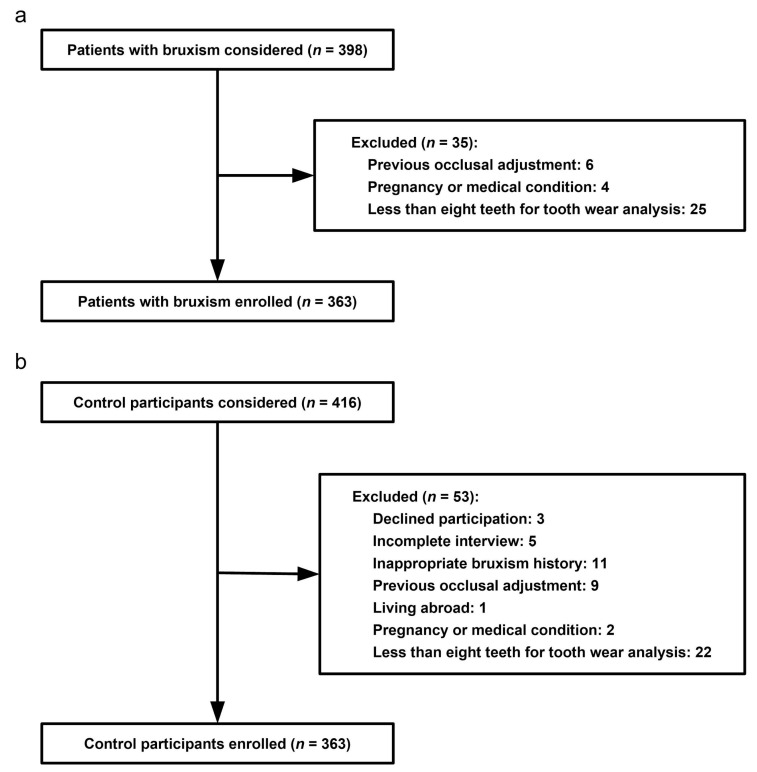
(**a**) Flow diagram of case subject enrollment; (**b**) flow diagram of control subject enrollment.

Of the 398 consecutive bruxism patients initially considered for the present study, 35 patients (8.8%) were excluded. We finally recruited 363 bruxism patients (91.2%). Details of case patient enrollment are described in Figure 1a. Among the 416 control subjects invited to participate, 53 (12.7%) were excluded. The remaining 363 participants (87.3%) were recruited as control participants. Details of control subject enrollment are described in Figure 1b.

Among the 363 case patients, 319 (87.9%) had sleep bruxism alone, 14 (3.9%) had awake bruxism alone, and 30 (8.3%) had both awake and sleep bruxism. Among these case patients, 329 (90.6%) had tooth wear into dentin (attrition exclusively: 136; attrition combined with erosion and/or abrasion: 193), 237 (65.3%) with temporal headaches, 17 (4.7%) with jaw locking when waking up, 192 (52.9%) with jaw muscle discomfort when waking up, and 189 (52.1%) with more than one symptoms. There were three missing data on cigarettes consumption of current smokers (control participants) and six missing data on body mass index (two case participants and four control participants). All the questionnaires were complete because of the close supervision. Table S1 displays the main characteristics of the 726 recruited participants.

Bruxism-related factors with *p*-value < 0.1 in the univariate analyses contained never married, depression, anxiety, pain-related temporomandibular disorders, high possibility of obstructive sleep apnea, and impaired sleep quality (Table S2). After adjusting for these confounding factors, GERD was associated with bruxism (odds ratio, 5.30; 95% confidence interval, 2.62–10.70; Model 1 in Table 1). Patients with a longer duration of GERD symptoms had a higher odds ratio for bruxism than those with a shorter duration (>5 versus ≤5 years; Model 1 in Table 1). The connection between bruxism and GERD remained significant despite identification of reduced association after additional adjustment for tooth wear in the sensitivity analysis (Model 2 in Table 1).

### 3.2. Cross-Sectional Study

All participants had occlusion and at least one surface with a tooth wear index score ≥1, which indicated wear into enamel and/or minimal loss of contour of cervical areas, or more extensive wear condition. Table 2 discloses the associations of severe tooth wear for the whole dentition with different types of bruxism by unconditional logistic regression. Among all the potential confounding factors examined, age ≥31 years, male gender, smoking, heavy drinking, tea consumption weekly or more, and acidic diet were positively associated with tooth wear, while timely rinsing (i.e., rinsing immediately after exposure to extrinsic/intrinsic acids) was inversely related to tooth wear in the univariate analyses (Table S3). After adjusting for these confounding factors, sleep bruxism as well as sleep and awake bruxism overlap were identified as independent risk factors for severe tooth wear, but not awake bruxism (Table 2).

Participants were regrouped as non-bruxism (*n* = 363), bruxism without GERD (*n* = 295), bruxism with GERD ≤5 years (*n* = 28) and bruxism with GERD >5 years (*n* = 40). The proportions of participants with tooth wear for the whole dentition and all tooth locations and surfaces, except for cervical areas, were different among these groups (Table S4).

Bruxism patients with GERD symptoms for a longer duration had a higher odds ratio for severe tooth wear for the whole dentition after adjustment (>5 versus ≤5 years; Table 3). Increased odds ratios for severe tooth wear were also identified in all tooth locations and occlusal/incisal and palatal/lingual surfaces, but not buccal/labial surfaces and cervical areas, in bruxism patients suffering from GERD for more than five years (Table 4, Table 5, Table 6 and Table 7).

Goodness-of-fit tests showed that the predicted values and the observed values were finely matched in each logistic regression model.

## 4. Discussion

The present case-control study indicates that GERD, especially long-term GERD, is a risk factor of bruxism. According to the companion cross-sectional study, GERD is associated with tooth wear in patients with bruxism. Hence, we have to reject the two null hypotheses. The present work shows, for the first time, that bruxism patients with GERD for extensive time-periods are prone to severe tooth wear.

Sleep bruxism was diagnosed by symptoms and signs using the American Academy of Sleep Medicine criteria in this study. Agreement between clinically-diagnosed and polysomnography-diagnosed sleep bruxism is debatable [3]. The findings of this study may not be generalizable to sleep bruxism diagnosed by polysomnography. The fluctuations of bruxism symptoms and signs and the unreliability of subjective response should be considered in the explication of the findings [3].

Several strengths outweigh the aforementioned weaknesses, including the ample study size that allowed for adjustment of potential confounding variables and the influential novelty of considering the relationship between GERD and bruxism in the evaluation of tooth wear.

The relationship between GERD and bruxism reported herein is in line with previous literature [9,10]. The relationship between GERD and bruxism has been discussed in detail previously [13]. This relationship is applicable to patients with awake bruxism alone as well as those with sleep bruxism alone [13]. Although evaluation of bruxism by polysomnography has superiority in providing objective profiles, diagnoses based on symptoms and signs have advantage in evaluating the clinical impact of the disorder [21]. This study focused on tooth wear, the most frequently-reported clinical impact of bruxism. The relationship between bruxism and tooth wear is contentious in the literature. In this study, among the consecutive patients reporting teeth grinding/clenching, more than 90% had at least one tooth with dentin wear. Severe tooth wear was related to sleep bruxism, while the relationship between severe tooth wear and awake bruxism was not significant. A previous study based on diagnostic casts suggested that tooth attrition was associated with self-reported bruxism [22]. Polysomnography evaluation also indicates that repetitive mighty grinding during sleep may lead to severe attrition [23]. Evidence on the relationship between tooth wear and awake bruxism exclusively is scarce. Awake bruxism is characterized by clenching, bracing or thrusting, which are not expected to cause tooth wear. Because only 3.9% of the case patients had awake bruxism alone, the finding that GERD was associated with intensified tooth wear may only be applicable to patients with sleep bruxism. Considering the relationship between GERD and bruxism, tooth wear in patients with sleep bruxism may be the consequence of attrition intensified by intrinsic acids rather than attrition alone. The finding that GERD was associated with intensified tooth wear into dentin in patients with sleep bruxism supports and advances the understanding that tooth wear is a multifarious condition involving multiple mechanisms [1,15]. Mechanical wear is enhanced by chemical wear caused by extrinsic or intrinsic acids [1]. Acids not only dissolve hard tissues directly, but also make tooth surfaces defenseless to mechanical wear [1]. Because of the presence of dentinal tubules and its higher organic content, dentin is more vulnerable to erosive agents than enamel [8]. Some proteolytic enzymes in dentin or saliva may be activated by acids and potentially degrade the organic substances in dentin [24]. For patients with GERD, apart from gastric acid, the proteases in the refluxate, such as pepsin and trypsin, also play a role in dentin wear [14].

Tooth wear in patients with GERD has also been associated with impaired salivary function [6]. Saliva is important for dilution and buffering of erosive agents and the development of the acquired pellicle, a perm-selective membrane layer on tooth surfaces that can protect the teeth from erosion [25]. Inferior salivary function has been found in patients with GERD, compared with healthy subjects [9]. The combination of parafunctional tooth-tooth contact, erosive refluxate, and impaired salivary function makes patients with both sleep bruxism and GERD more susceptible to tooth wear. The hypothesized associations among GERD, saliva, sleep bruxism, and tooth wear deserve further investigation.

**Figure 2:**
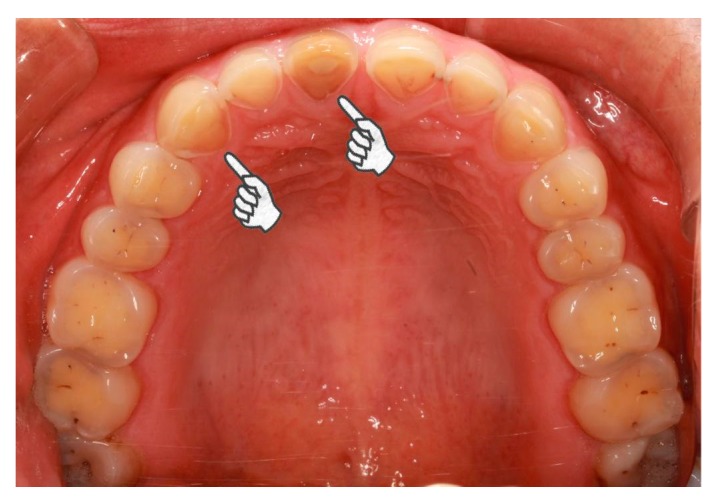
Pathological tooth wear of a 23-year-old female case participant. In general, the female is healthy but she suffers from daily regurgitation and/or heartburn for about two years. She complained of teeth grinding during sleep every day. She rarely experiences exposure to acids from the environment or diet. She hardly rinses or brushes after regurgitation. Extensive tooth wear into dentin was identified, especially on the palatal superficies of upper incisors and canines (pointers).

The present study identified an increased odds ratio for severe tooth wear in bruxism patients with GERD for an extensive time period. The influence of erosive agents on tooth wear is intensified by extended time-periods [26]. Biological, chemical, and behavioral factors interact with the tooth surfaces, wearing the hard tissue off over time [19]. When the association between severe tooth wear and GERD in bruxism patients was evaluated by different tooth locations and surfaces, the association was significant in all tooth locations and the palatal/lingual and occlusal/incisal surfaces but not the buccal/labial surfaces and cervical areas. Although the number of bruxism patients with GERD was small, the findings were convincing because of the calibration of the regression models. The areas of tooth erosion caused by intrinsic acids have characteristic distribution. Erosive refluxate initially attacks the palatal superficies of the upper teeth, especially the upper incisors due to the absence of protection from the major salivary glands [14]. At first, the lower teeth were protected by the tongue [14]. However, when the acid challenge continues, erosion occurs in the posterior lower teeth, beginning with the lingual, subsequently the occlusal, and eventually the buccal surfaces [14]. The combined action of attrition, erosion and abrasion may result in extensive tooth wear, even in young people (Figure 2). The high prevalence of erosive tooth wear in Western populations [27] makes the prevention and management of tooth wear more critical. It is worthwhile to investigate the associations among bruxism, GERD, and tooth wear on Western populations.

Due to limitations of the case-control and cross-sectional designs, causal relationships cannot be established in the present work. The relationship between GERD and bruxism may be bidirectional, making the mechanisms of tooth wear more complicated. Advanced erosive wear is also associated with pathological reflux in the absence of reflux symptoms [28]. The associations among asymptomatic GERD, bruxism, and tooth wear are worthy of further research. Clinical trials are required to determine whether the management of GERD would have an effect on bruxism.

The underlying mechanisms of cervical wear are controversial [2]. The present study found no evidence linking cervical wear to bruxism or GERD. The results are contrary to some findings [22,29], but consistent with the other findings [30,31].

## 5. Conclusions

The present work identifies a relationship between GERD and bruxism. Special attention should be devoted to this association because both conditions are related to tooth wear. One condition may aggravate the other, resulting in extensive tooth wear. As identified in the present study, long-term GERD is associated with severe tooth wear in bruxism patients. These findings provide important insights on the mechanism of tooth wear in bruxism patients. More importantly, it is time to change the conventional perspective that regards GERD and bruxism as two separate factors associated with tooth wear. Integrative medicine emphasizes that the well-being of the whole person is critical in the prevention of chronic diseases [32]. Dentists are always the first to notice tooth wear in bruxism patients. Any sign of pathological tooth wear due to erosion and/or attrition in bruxism patients should elicit further investigations on reflux symptoms. It is important for dentists to conduct a thorough evaluation of dietary habits, oral hygiene, and medical conditions including GERD to detect the etiological factors of tooth wear in bruxism patients.

## Figures and Tables

**Table 1:** Association between bruxism and GERD using conditional logistic regression.

Variables	Bruxism (*n* = 363) ^1^	Non-Bruxism (*n* = 363) ^1^	Univariate Analysis		Multivariable Analysis			
					Model 1 ^2^		Model 2 ^3^	
			OR (95% CI)	*p*-Value	OR (95% CI)	*p*-Value	OR (95% CI)	*p*-Value
GERD	68 (18.7)	13 (3.6)	6.21 (3.36–11.46)	<0.001	5.30 (2.62–10.70)	<0.001	3.84 (1.86–7.95)	<0.001
GERD duration								
≤5 years	28 (7.7)	8 (2.2)	4.15 (1.86–9.25)	<0.001	3.38 (1.38–8.24)	0.008	2.49 (0.98–6.29)	0.054
>5 years	40 (11.0)	5 (1.4)	9.49 (3.70–24.36)	<0.001	8.73 (2.97–25.63)	<0.001	6.27 (2.07–18.97)	0.001

Abbreviations: CI, confidence interval; GERD, gastroesophageal reflux disease; OR, odds ratio. ^1^ Values listed in these columns are numbers (percentages) of subjects. ^2^ Adjusted for marital status, pain-related temporomandibular disorder, possibility of obstructive sleep apnea, sleep quality, depression and anxiety. ^3^ Adjusted for marital status, pain-related temporomandibular disorder, possibility of obstructive sleep apnea, sleep quality, depression, anxiety, and tooth wear.

**Table 2:** Associations of severe tooth wear for the whole dentition with different types of bruxism using binary logistic regression.

Variables	With Severe Tooth Wear (*n* = 224) ^1^	Without Severe Tooth Wear (*n* = 502) ^1^	Univariate Analysis		Multivariable Analysis ^2^	
			OR (95% CI)	*p*-Value	OR (95% CI)	*p*-Value
Non-bruxism	76 (33.9)	287 (57.2)	Reference		Reference	
Sleep bruxism	128 (57.1)	191 (38.0)	2.53 (1.81–3.55)	<0.001	2.77 (1.82–4.21)	<0.001
Awake bruxism	6 (2.7)	8 (1.6)	2.83 (0.95–8.41)	0.061	1.98 (0.55–7.17)	0.296
Overlap of sleep and awake bruxism	14 (6.3)	16 (3.2)	3.30 (1.54–7.07)	0.002	3.28 (1.26–8.54)	0.015

Abbreviations: CI, confidence interval. ^1^ Values listed in these columns are numbers (percentages) of subjects. ^2^ Adjusted for age, sex, gastroesophageal reflux disease, smoking, heavy drinking, tea consumption, acidic diet, and timely rinsing.

**Table 3:** Associations among bruxism, GERD, and severe tooth wear for the whole dentition.

Variables	With Severe Tooth Wear (*n* = 224) ^1^	Without Severe Tooth Wear (*n* = 502) ^1^	Univariate Analysis		Multivariable Analysis ^2^	
			OR (95% CI)	*p*-Value	OR (95% CI)	*p*-Value
Non-bruxism	76 (33.9)	287 (57.2)	Reference		Reference	
Bruxism without GERD	115 (51.3)	180 (35.9)	2.41 (1.71–3.51)	<0.001	2.89 (1.90–4.39)	<0.001
Bruxism with GERD ≤5 years	10 (4.5)	18 (3.6)	2.10 (0.93–4.73)	0.074	2.04 (0.77–5.43)	0.153
Bruxism with GERD >5 years	23 (10.3)	17 (3.4)	5.11 (2.60–10.04)	<0.001	4.70 (2.04–10.83)	<0.001

Abbreviations: CI, confidence interval; GERD, gastroesophageal reflux disease; OR, odds ratio. ^1^ Values listed in these columns are numbers (percentages) of subjects. ^2^ Adjusted for age, sex, smoking, heavy drinking, tea consumption, acidic diet, and timely rinsing.

**Table 4:** Associations among bruxism, GERD, and severe tooth wear for different tooth surfaces in the univariate analysis.

Variables	Occlusal/Incisal		Palatal/Lingual		Buccal/Labial		Cervical	
	OR (95% CI)	*p*-Value	OR (95% CI)	*p*-Value	OR (95% CI)	*p*-Value	OR (95% CI)	*p*-Value
Non-bruxism	Reference		Reference		Reference		Reference	
Bruxism without GERD	2.47 (1.74–3.49)	<0.001	3.30 (1.16–9.37)	0.025	1.78 (0.67–4.75)	0.246	1.03 (0.44–2.41)	0.952
Bruxism with GERD ≤5 years	2.21 (0.98–4.98)	0.057	8.59 (1.94–38.04)	0.005	1.78 (0.22–15.87)	0.560	2.25 (0.48–10.59)	0.305
Bruxism with GERD >5 years	4.86 (2.48–9.52)	<0.001	15.19 (4.57–50.51)	<0.001	1.30 (0.16–10.88)	0.806	2.37 (0.64–8.79)	0.196

Abbreviations: CI, confidence interval; GERD, gastroesophageal reflux disease; OR, odds ratio.

**Table 5:** Associations among bruxism, GERD, and severe tooth wear for different tooth surfaces after adjustment ^1^.

Variables	Occlusal/Incisal		Palatal/Lingual	
	OR (95% CI)	*p*-Value	OR (95% CI)	*p*-Value
Non-bruxism	Reference		Reference	
Bruxism without GERD	2.92 (1.92–4.45)	<0.001	2.72 (0.92–8.10)	0.072
Bruxism with GERD ≤5 years	2.15 (0.81–5.74)	0.125	8.52 (1.66–43.71)	0.010
Bruxism with GERD >5 years	4.23 (1.84–9.75)	0.001	8.57 (2.82–32.21)	0.001

Abbreviations: CI, confidence interval; GERD, gastroesophageal reflux disease; OR, odds ratio. ^1^ Adjusted for age, sex, smoking, heavy drinking, tea consumption, acidic diet, and timely rinsing.

**Table 6:** Associations among bruxism, GERD, and severe tooth wear for different tooth locations in the univariate analysis.

Variables	Upper		Lower		Anterior		Posterior	
	OR (95% CI)	*p*-Value	OR (95% CI)	*p*-Value	OR (95% CI)	*p*-Value	OR (95% CI)	*p*-Value
Non-bruxism	Reference		Reference		Reference		Reference	
Bruxism without GERD	2.57 (1.75–3.76)	<0.001	2.33 (1.63–3.32)	<0.001	2.63 (1.77–3.92)	<0.001	2.08 (1.44–2.99)	<0.001
Bruxism with GERD ≤5 years	1.95 (0.79–4.81)	0.148	2.06 (0.89–4.74)	0.091	3.19 (1.36–7.45)	0.008	2.17 (0.94–5.02)	0.069
Bruxism with GERD >5 years	5.29 (2.67–10.50)	<0.001	4.34 (2.21–8.51)	<0.001	4.97 (2.47–9.99)	<0.001	4.59 (2.33–9.01)	<0.001

Abbreviations: CI, confidence interval; GERD, gastroesophageal reflux disease; OR, odds ratio.

**Table 7:** Associations among bruxism, GERD, and severe tooth wear for different tooth locations after adjustment ^1^.

Variables	Upper		Lower		Anterior		Posterior	
	OR (95% CI)	*p*-Value	OR (95% CI)	*p*-Value	OR (95% CI)	*p*-Value	OR (95% CI)	*p*-Value
Non-bruxism	Reference		Reference		Reference		Reference	
Bruxism without GERD	2.61 (1.67–4.06)	<0.001	2.68 (1.75–4.11)	<0.001	2.66 (1.68–4.21)	<0.001	2.18 (1.42–3.36)	<0.001
Bruxism with GERD ≤5 years	1.55 (0.55–4.37)	0.413	2.02 (0.74–5.51)	0.171	3.10 (1.14–8.45)	0.027	2.06 (0.77–5.55)	0.153
Bruxism with GERD >5 years	3.74 (1.63–8.58)	0.002	3.71 (1.60–8.61)	0.002	3.16 (1.37–7.32)	0.007	3.92 (1.69–9.05)	0.001

Abbreviations: CI, confidence interval; GERD, gastroesophageal reflux disease; OR, odds ratio. ^1^ Adjusted for age, sex, smoking, heavy drinking, tea consumption, acidic diet, and timely rinsing.

## Supplementary Materials

The following are available online at http://www.mdpi.com/2077-0383/7/11/417/s1, Table S1: Characteristics of cases (with bruxism) and controls (without bruxism), Table S2: Factors associated with bruxism, Table S3: Factors associated with severe tooth wear, Table S4: Conditions of tooth wear for different subgroups.
