# Supplementary material for: Associations among Bruxism, Gastroesophageal Reflux Disease, and Tooth Wear
Source: J Clin Med. 2018 Nov 6;7(11):417. doi: 10.3390/jcm7110417 (PMC6262381; doi:10.3390/jcm7110417)
Supplement: Supplementary file 1 [file jcm-07-00417-s001.pdf]

SUPPLEMENTARY MATERIALS

TABLE OF CONTENTS

Table S1. Characteristics of cases (with bruxism) and controls (without bruxism)....................................2

Table S2. Factors associated with bruxism...3

Table S3. Factors associated with severe tooth wear...4

Table S4. Conditions of tooth wear for different subgroups...5

Table S1. Characteristics of cases (with bruxism) and controls (without bruxism).

Characteristic	Cases (n = 363)[1]	Controls (n = 363)[1]	*P*
**Age, median (IQR), years**	28 (22 - 38)	29 (23 - 39)	0.720
**Sex**			> 0.99
Male	147 (40.5)	147 (40.5)	
Female	216 (59.5)	216 (59.5)	
**Residence**			0.696
Urban	241 (66.4)	236 (65.0)	
Rural	122 (33.6)	127 (35.0)	
**BMI, median (IQR), kg/m²**	21.4 (19.4 - 23.5)	21.0 (19.0 - 23.7)	0.833
**Obesity**	8 (2.2)	6 (1.7)	0.589
**Marital status**			0.083
Single	180 (49.6)	151 (41.6)	
Married	177 (48.8)	207 (57.0)	
Divorced/widowed	6 (1.7)	5 (1.4)	
**Education level**			0.200
≤ 9 years	37 (10.2)	38 (10.5)	
> 9 - 12 years	47 (12.9)	64 (17.6)	
> 12 years	279 (76.9)	261 (71.9)	
**Smoking status**			0.091
Never	280 (77.1)	301 (82.9)	
Former	22 (6.1)	12 (3.3)	
Current	61 (16.8)	50 (13.8)	
No. cigarettes/day[2], median (IQR)	10 (5 - 15)	10 (6 - 20)	0.789
**Alcohol**			0.582
None/moderate	285 (78.5)	291 (80.2)	
Heavy	78 (21.5)	72 (19.8)	
**Tea**			0.693
None/less than weekly	214 (59.0)	204 (56.2)	
Weekly	96 (26.4)	106 (29.2)	
Daily	53 (14.6)	53 (14.6)	
**Coffee**			0.955
None/less than weekly	309 (85.1)	308 (84.8)	
Weekly	49 (13.5)	49 (13.5)	
Daily	5 (1.4)	6 (1.7)	
**Hypertension**	12 (3.3)	11 (3.0)	0.832

Abbreviations: BMI, body mass index; IQR, interquartile range.

[1] Values listed in these columns are numbers (percentages) of subjects.

[2] Consumption of current smokers.

Table S2. Factors associated with bruxism.

**Variables**	**Bruxism (n = 363)**	**Non-bruxism (n = 363)**	***P***
Never married	180 (49.6)	151 (41.6)	0.031
High likelihood of obstructive sleep apnea	43 (11.8)	24 (6.6)	0.015
Pain-related temporomandibular disorder	80 (22.0)	58 (16.0)	0.037
Depression	71 (19.6)	23 (6.3)	< 0.001
Anxiety	41 (11.3)	18 (5.0)	0.002
Impaired sleep quality	180 (49.6)	132 (36.4)	< 0.001

Values are numbers (percentages) of subjects.

Table S3. Factors associated with severe tooth wear.

Variables	With severe tooth wear (n = 224)[1]	Without severe tooth wear (n = 502)[1]	*P*
Age ≥ 31 years	174 (77.7)	140 (27.9)	< 0.001
Male	110 (49.1)	184 (36.7)	0.002
Current smoker	52 (23.2)	59 (11.8)	< 0.001
Heavy drinker[2]	62 (27.7)	88 (17.5)	0.002
Tea consumption[3]	113 (50.4)	195 (38.8)	0.003
Acidic diet[4]	93 (41.5)	129 (25.7)	< 0.001
Timely rinsing[5]	3 (1.3)	23 (4.6)	0.030

Abbreviations: CI, confidence interval; GERD, gastroesophageal reflux disease; OR, odds ratio.

[1] Values listed in these columns are numbers (percentages) of subjects.

[2] Had unsafe drinking (> 15 grams/day for females and > 25 grams/day for males) at least one day a week.

[3] Weekly or more.

[4] Daily or more consumption of acidic foods.

[5] Rinsing immediately after exposure to intrinsic or extrinsic acids.

Table S4. Conditions of tooth wear for different subgroups.

Variables	Non-bruxism (n = 363)	Bruxism without GERD (n = 295)	Bruxism with GERD ≤ 5 years (n = 28)	Bruxism with GERD > 5 years (n = 40)	*P*
The whole dentition	253 (69.7)	262 (88.8)	27 (96.4)	40 (100.0)	< 0.001
SURFACE					
Occlusal/incisal	246 (67.8)	261 (88.5)	27 (96.4)	40 (100.0)	< 0.001
Palatal/lingual	90 (24.8)	151 (51.2)	21 (75.0)	29 (72.5)	< 0.001
Buccal/labial	45 (12.4)	69 (23.4)	8 (28.6)	11 (27.5)	< 0.001
Cervical	19 (5.2)	22 (7.5)	2 (7.1)	4 (10.0)	0.525
LOCATION					
Upper	216 (59.5)	249 (84.4)	27 (96.4)	39 (97.5)	< 0.001
Lower	237 (65.3)	248 (84.1)	27 (96.4)	40 (100.0)	< 0.001
Anterior	217 (59.8)	241 (81.7)	26 (92.9)	40 (100.0)	< 0.001
Posterior	212 (58.4)	238 (80.7)	25 (89.3)	40 (100.0)	< 0.001

Abbreviation: GERD, gastroesophageal reflux disease.

Values are numbers (percentages) of subjects with tooth wear into dentin.
